# Pyogenic Brain Abscess Caused by *Peptostreptococcus* in a Patient with HIV-1 Infection

**DOI:** 10.3390/diseases5040026

**Published:** 2017-11-17

**Authors:** Jose Armando Gonzales Zamora, Luis Alberto Espinoza

**Affiliations:** 1 Division of Infectious Diseases, Department of Medicine, University of Miami, Miller School of Medicine, Miami, FL 33136, USA; 2 Gilead Sciences, Miami, FL 33136, USA

**Keywords:** pyogenic brain abscess, *Peptostreptococcus*, HIV, stereotactic needle aspiration

## Abstract

In the setting of HIV, cerebral lesions are usually secondary to lymphoma and opportunistic infections; however, in patients with CD4 counts above 200 cells/uL, other pathologies such as pyogenic brain abscess could gain importance. The microbiology of pyogenic brain abscess has *Staphylococcus* and *Streptococcus* as the leading etiologic pathogens in immunocompetent individuals. *Peptostreptococcus* is also recognized as a common cause of brain abscess in this patient population. In HIV-infected individuals, there have been sporadic reports of *Peptostreptococcus* infections but none of brain abscess. We describe the case of a 43-years-old HIV-infected patient with a CD4 count of 350 cells/uL that developed a *Peptostreptococcus* brain abscess presumably from hematogenous spread of an odontogenic source. Treatment with stereotactic needle aspiration in two opportunities and four weeks of intravenous antibiotics led to a complete resolution of this infection. This case highlights the importance of a multidisciplinary approach for an effective treatment of pyogenic brain abscess in HIV-1 patients.

## 1. Introduction

Lymphoma and opportunistic infections have historically been the leading causes of cerebral lesions in patients with advanced HIV; however, the introduction of highly active antiretroviral therapy has led to a radical change in the natural history of HIV and made way to different malignant processes and infections not commonly associated with immunosuppression [1]. Pyogenic brain abscess is one of these multiple pathologies, and although its epidemiology and microbiology is well known in immunocompetent hosts, the literature is scarce in HIV-infected patients. One of the most important etiologic pathogens of pyogenic brain abscess in immunocompetent individuals is *Peptostreptococcus*, but no cases have been described in the context of HIV [2]. In this report, we present the first case of *Peptostreptococcus* causing pyogenic brain abscess in a patient with HIV-1 infection. We also discuss the epidemiology and pathophysiology of pyogenic brain abscess, along with the multidisciplinary approach that its treatment should carry.

## 2. Case Description

**Figure 1:**
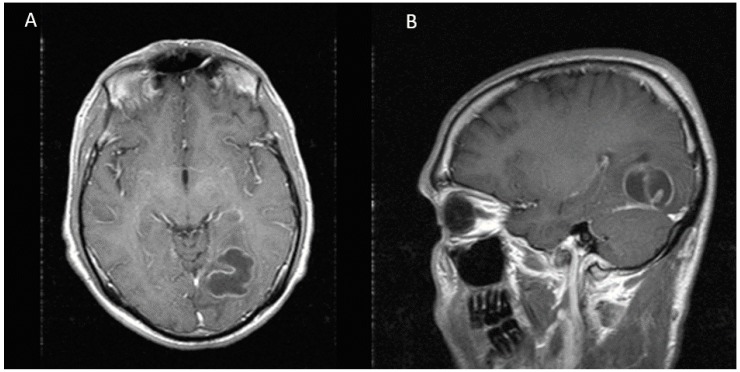
MRI showing a multilobulated and multiseptated lesion of 31 × 31 × 26 mm with peripheral enhancement in the left occipital lobe. (**A**) Axial view (**B**) Sagittal view.

**Figure 2:**
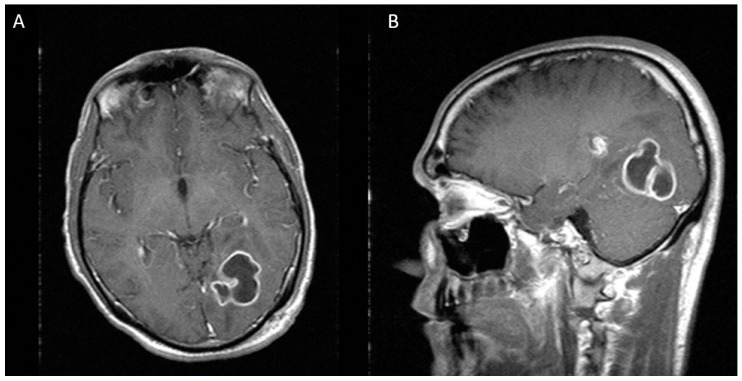
Two weeks after first stereotactic needle aspiration, MRI demonstrates an increase in the abscess size, now measuring 36 × 33 × 40 mm. (**A**) Axial view (**B**) Sagittal view.

**Figure 3:**
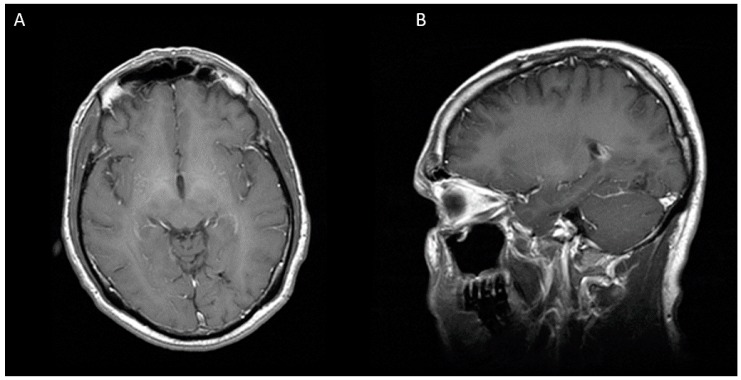
Eight months after second stereotactic needle aspiration, MRI is showing a complete resolution of cerebral abscess. (**A**) Axial view (**B**) Sagittal view.

A 43-years-old white Hispanic male presented to the hospital with frontal headaches, sore throat, rhinorrhea, and subjective fevers in the last three days. He denied any sick contacts or recent travels. His past medical history was significant for HIV-1 infection and chronic hepatitis C. His antiretroviral therapy consisted of tenofovir/emtricitabine, atazanavir, and ritonavir. He admitted not to be fully compliant with his treatment. His most recent CD4 count and viral load were 350 cells/uL and 4727 copies/mL, respectively. On admission, his vitals were within normal limits. Neurologic examination was remarkable for right superior quadrantonopia. Extraocular movements were intact. No other focal or meningeal signs were noted. Lung examination was normal. Laboratory studies were significant for normal white blood cell count (6.9 K/uL), low platelet count (85 K/uL), low serum sodium (132 mm/L), normal creatinine (0.73 mg/dL), elevated transaminases (AST 152 units/L and ALT 93 units/L), and elevated total bilirubin (2.4 mg/dL). Given the abnormal neurologic findings, a brain computed tomography scan (CT scan) was ordered, which showed a mixed hypo and isodense intraparenchymal lesion in the left occipital lobe with mass effect on the occipital horn of the left lateral ventricle. No sinusitis or mastoiditis was noted. To better characterize this lesion, a magnetic resonance imaging (MRI) was performed. This image showed a multilobulated and multiseptated mass of 31 × 31 × 26 mm with peripheral enhancement and a cystic necrotic center (Figure 1). These findings raised the concern for a malignancy or an opportunistic infection such as toxoplasmosis. The workup for malignancy included an abdominal ultrasound and a computed tomography of chest, abdomen, and pelvis. All of them were unremarkable. The patient was started on empiric toxoplasmosis therapy with pyrimethamine, sulfadiazine, and leucovorin. The serology for toxoplasmosis came back negative. On hospital day 8, the patient underwent stereotactic biopsy of the cerebral lesion. Ten milliliters of purulent fluid were aspirated and sent for culture. Additionally, two tissue samples were obtained and sent for pathology. Smear showed many white blood cells and gram-positive cocci in chains. The patient was started on ceftriaxone and metronidazole, and treatment for toxoplasmosis was discontinued. He also received levaritacetam for seizure prophylaxis. The biopsy revealed acute inflammatory changes with a focus of necrosis, but no malignant cells were noted. On hospital day 11, cultures from purulent fluid yielded heavy growth of *Peptostreptococcus* sp. Identification was confirmed by Microscan Walkaway 96 (Sacramento, CA, USA). No susceptibility test was done. To evaluate for a distant source of infection, an echocardiogram and a panorex X-ray were ordered. The echocardiogram was negative for vegetations, but the panorex X-ray revealed a cavity in the second tooth without abscess formation. The plan was to repeat CT scan after two weeks of antibiotic therapy to assess for treatment response. Unfortunately, the patient’s mental status deteriorated, and a repeat CT scan demonstrated an increase in brain abscess size, now measuring 36 × 33 × 40 mm (Figure 2). For this reason, we decided to perform a second needle aspiration. A stereotactic approach was preferred over radical resection, given the proximity of the lesion to critical visual pathways that carried a high risk of permanent damage. On hospital day 23, he underwent repeat stereotactic aspiration of the abscess and approximately 7 cc of purulent material was evacuated. On hospital day 25, antibiotic regimen was switched to high dose penicillin due to the concern for metronidazole resistance and he completed a total course of four weeks of intravenous antibiotics. The patient was discharged in stable condition with normal mentation and minimal visual deficits. At eight-month follow-up, neurologic examination revealed total resolution of visual deficits and MRI did not show any evidence of brain abscess (Figure 3).

## 3. Discussion

Brain abscess is a focal suppurative process of the brain parenchyma, and although relatively uncommon, it continues to be an important cause of morbidity and mortality even with the advancement of imaging technologies and antibiotic therapy [3]. The estimated incidence of brain abscesses is 0.9 per 100,000 person-years in developed countries with an overall mortality rate that can reach up to 32% [3,4]. A review by Xiao et al. reported a male predominance (male to female ratio 2.7/1) and a mean age at presentation of 43 ± 21 years, all of which corresponds to our patient demographic data [4].

The mechanisms by which bacteria invade the brain parenchyma are diverse and include spread from a contiguous focus of infection (e.g., sinusitis, otitis media, and mastoiditis), direct inoculation (e.g., trauma and neurosurgery), and hematogenous seeding from a distant focus. In this latter category, distant infections reported as potential sources include pulmonary, skin, abdominal and pelvic infections as well as endocarditis. According to some reports, the leading pathophysiologic mechanism for brain abscess development is direct spread from a contiguous focus, which represents 40–50% of the cases, followed by hematogenous seeding in 25% of the cases [5]. Our patient work-up to search for an infectious source was unremarkable, except for the finding of a second tooth cavity without abscess formation. We hypothesize that our patient developed a brain abscess by hematogenous spread from an odontogenic origin, which is an uncommonly reported event [6].

Immunosuppression is a highly recognized predisposing condition for brain abscesses. In a review published by Xiao et al. that included 178 patients, immunosuppression was present in 11.8% of the cases. In this study, 10 patients (5.6%) had HIV/AIDS, 10 patients (5.6%) suffered from a hematologic condition, and one patient (0.6%) was on steroids. Immunosuppression was also an independent factor for poor outcome. An interesting finding of this study was the high mortality rate (75%) in immunocompromised individuals [4].

The differential diagnosis of brain lesions in HIV patients is diverse and depends on the degree of immunosuppression. In patients with CD4 count <200 cells/uL, the leading diagnostic considerations are toxoplasmosis, primary central nervous system lymphoma, and progressive multifocal leukoencephalopathy (PML). However, in patients with a CD4 count above 200 cells/uL, benign and malignant tumors predominate as in immunocompetent individuals. The frequency of pyogenic brain abscesses in patients with well-controlled HIV and high CD4 cell counts is unknown. The literature is very limited in this regard, with only a few cases reported [7,8]. It is unclear if HIV infection by itself increases the risk of brain abscesses. In this respect, some authors such as Li et al. have described the presence of persistent chronic inflammation secondary to shedding of the HIV Tat protein, which can affect the integrity of the blood-brain barrier, even with ongoing antiretroviral therapy [9]. This raises the possibility of bacteria gaining access to the brain parenchyma through altered brain-blood barrier as a complication of HIV infection.

There is a wide range of pathogens that can cause pyogenic brain abscess. A systematic review published by Brouwer et al. revealed that *Streptococcus* and *Staphylococcus spp* are the most frequently isolated organisms [2]. Among these species, *Streptococcus viridans* and *Staphylococcus aureus* are the commonest strains. In recent reviews, anaerobes constitute one of the most important isolated organisms, with a frequency rate that can reach up to 40%; being *Peptostreptococcus* the species most frequently isolated (54% of cases) [10]. The majority of anaerobic abscesses occur by contiguous spread from the upper respiratory tract and oral cavity, where anaerobes are found as part of the commensal flora. In our patient, this supports the theory of a dental cavity as a potential source of *Peptostreptococcus* that led to hematogenous spread and invasion of the brain parenchyma.

Unlike immunocompetent hosts, the microbiology of pyogenic brain abscesses in HIV patients with CD4 count >200 cells/uL has not been well defined. The literature is very limited, with only anecdotal reports of *Staphylococcus*, *Propionibacterium*, and *Streptococcus* as some of the pathogens isolated [7,8]. In the setting of HIV, *Peptostreptococcus* has been associated with skin abscesses, periodontitis, and pneumonia, but no cases of pyogenic brain abscess have been reported [11,12,13].

The treatment of pyogenic brain abscess requires a multidisciplinary approach that includes use of antibiotics, neuro-radiologic evaluation and surgical intervention. Initial therapy should be commenced with broad-spectrum antibiotics, which cross blood-brain and blood-CSF barriers in adequate concentrations. An empiric antimicrobial regimen should be based on the presumptive source and gram stain if available. For patients with an abscess arising from an oral, otogenic, or sinus source, treatment with ceftriaxone and metronidazole is recommended. Vancomycin is added when a hematogenous spread is suspected [14]. This empiric antibiotic regimen should be simplified as soon as the etiologic pathogen is identified by cultures. Our patient culture grew *Peptostreptococcus*, which is usually susceptible to penicillin, cephalosporins, carbapenems, and metronidazole. However, some cases of metronidazole resistance have been reported [15]. The duration of antibiotics is prolonged, usually four to eight weeks [14].

Surgery is largely required for treatment and diagnostic purposes. Needle aspiration and surgical excision are the two surgical options contemplated in these cases. Stereotactic needle aspiration is generally preferred given the lower rates of neurologic sequel [16]. Craniotomy is nowadays infrequently performed and is reserved for cases of traumatic or multiloculated brain abscess and in patients with clinical deterioration and radiographic progression despite needle aspiration [17]. We have to be cognizant that there are no pragmatic rules in the treatment of brain abscess and each case should be individualized and treated on its own merits. Our patient is a clear illustration of that. After an initial stereotactic needle aspiration, he worsened clinically and radiographically, but instead of a craniotomy with radical resection, we decided to perform a second needle aspiration, which along with four weeks of antibiotics, led to a complete resolution of the abscess.

## 4. Conclusions

Although rare, pyogenic brain abscess should be included in the differential diagnosis of brain lesions in HIV-1 patients with high CD4 counts. This is the first report of *Peptostreptococcus* pyogenic abscess in the setting of HIV infection, which resulted potentially from hematogenous spread of an odontogenic focus. The management of this infection is multidisciplinary and includes intravenous antibiotics, neuro-radiologic evaluation, and surgical intervention. Stereotactic needle aspiration seems to be treatment of choice, especially in cases where radical resection carries a high risk of permanent damage of neuro-optical tracts. More studies are needed to evaluate the epidemiology and microbiology of brain abscesses in the era of highly active antiretroviral therapy.
